# Familial hemophagocytic lymphohistiocytosis in a girl with a novel homozygous mutation of *STX11*: A case report

**DOI:** 10.1097/MD.0000000000018107

**Published:** 2019-11-27

**Authors:** Xia Guo, Mingyan Jiang, Xue Tang, Qiang Li

**Affiliations:** Department of Pediatric Hematology/Oncology and Key Laboratory of Birth Defect and Related Disorders of Women and Children (Sichuan University), Ministry of Education, West China Second University Hospital, Sichuan University, Chengdu, Sichuan, China.

**Keywords:** CD107a degranulation function, familial hemophagocytic lymphohistiocytosis, *STX11* gene

## Abstract

**Rationale:**

Familial hemophagocytic lymphohistiocytosis (FHL) is a rare fatal autosomal recessively inherited disease and can be divided into five types. The mortality of untreated patients is up to 95% and it can be healed only after immunochemotherapy for disease control and hematopoietic stem cell transplantation. Clinical data of a girl with late-onset and recurrent hemophagocytic lymphohistiocytosis (HLH) was retrospectively analyzed to determine the etiology and potential pathogenic gene.

**Patient concerns and clinical findings:**

The proband was a female child patient from a consanguineous marriage family who was 11 years old, and clinically manifested delayed (onset at the age of 4 years and 6 months) and recrudescent HLH. Both of her elder brothers died at the ages of 4 and 5 years, respectively. The patient had a degranulation function defect of CD107a in natural killer (NK) cells, and the degranulation function of cytotoxic T lymphocytes (CTL) obviously declined (ΔMFI: 1.4%, normal ≧2.8%); the degranulation function of NK cells and CTL of her father was also obviously reduced. To identify possible underlying genetic causes, gene mutation analysis was undertaken. A novel homozygous nonsense mutation in *STX11* (c.49C>T, p.Q17X) was documented, arising from both her parents.

**Diagnosis:**

According to the clinical manifestations and detection results of *STX11*, the diagnosis of FHL-type 4 was confirmed and her parents were heterozygotic carriers.

**Interventions and outcomes:**

Good responses to HLH-2004 chemotherapy had been achieved for each onset, and the maximum remission duration (without taking any drug) was 23 months. The patient has been alive for 82 months since the onset, and has stopped taking dexamethasone and etoposide, but is still on oral cyclosporine to maintain the treatment. She has performed HLA matching and now is actively looking for a donor to prepare hematopoietic stem cell transplantation.

**Conclusions:**

Relevant gene detections should be implemented at the earliest for young patients from consanguineous marriages and with a family history of HLH so as to provide a basis for etiological diagnosis and radical treatment by hematopoietic stem cell transplantation and provide accurate genetic counseling for family members.

## 1 Introduction

Familial hemophagocytic lymphohistiocytosis (FHL) is a rare fatal autosomal recessive disease, being clinically characterized by recurrent fever, hepatosplenomegaly, cytopenia, hypertriglyceridemia, and/or hypofibrinogenemia, obvious elevation of serum ferritins and hemophagocytosis in the bone marrow. Its pathogenesis is that the innate functional defect of perforin-dependent cytotoxicity in cytotoxic T cells (CTL)/natural killer (NK) cells causes them to fail to effectively remove abnormal cells with antigens, which further incurs the persistent and uncontrolled proliferation and activation of polyclonal T lymphocytes (mainly CD8^+^ T cells) and macrophages. These infiltrate solid organs such as the liver, spleen, lymph nodes, and bone marrow, generating an inflammatory cytokine storm and serious tissue necrosis.^[1–3]^ The mortality of untreated FHL is up to 95%.^[2]^ FHL can be divided into five types. In FHL-1 no related pathogenic gene has been found so far; four other types arise from the mutation of *PRF1* (FHL2)*, UNC13D* (FHL3), *STX11* (FHL4), and *STXBP2* (FHL5), respectively. Familial hemophagocytic lymphohistiocytosis usually presents at a very young age; in 10% of patients during their neonatal period, in 70% to 80% of patients before they are 1 year old and in 90% of patients before they are 2 years old. Only the minority come down with the illness during their puberty or even after reaching adulthood.^[2,4]^ In the cases registered by the International Histiocyte Society, only 5% of patients are attacked after they are 5 years old.^[2]^ This study reports a female child patient who presented with late-onset and recurrent hemophagocytic lymphohistiocytosis (HLH) arising from a new homozygous mutation of *STX11*; the relevant literature is reviewed.

## 2 Case report

**Figure 1:**
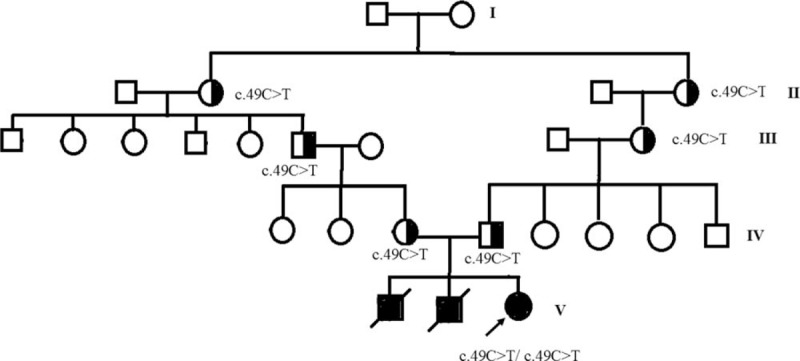
Pedigree of the proband. Her mother's grandmother and father's grandmother were sisters, and her two brothers died early.

**Figure 2:**
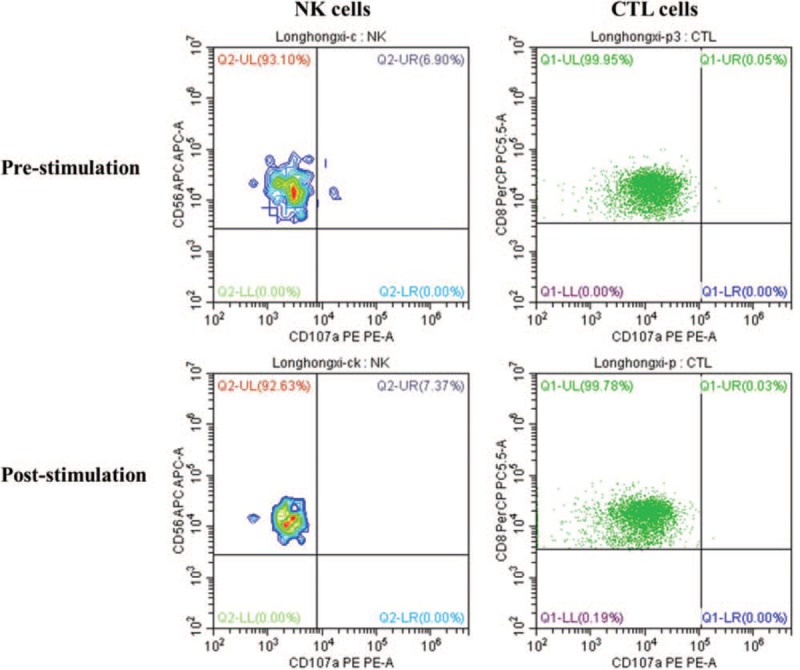
Analysis of the CD107a degranulation function of the proband. The CD107a degranulation function of the proband was obviously declined in cytotoxic T lymphocytes (ΔMFI: 1.4%, normal ≥2.8%) and NK cells (ΔCD107a = 0.47%, normal >10%).

**Figure 3:**
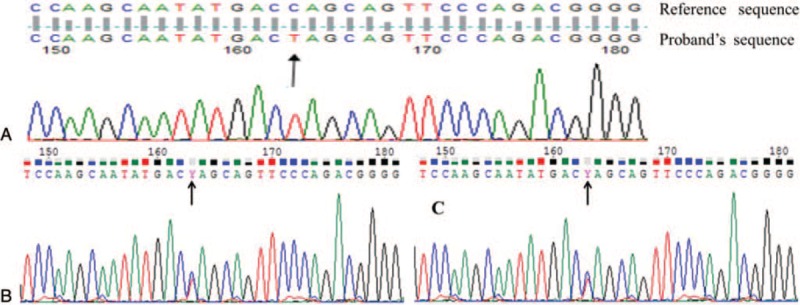
Results of *STX11* sequence analysis in the pedigree. (A) A homozygous mutation in *STX11* (c.49C>T) was demonstrated, which converted the codon encoding the glutamine amino acid at position 17 in syntaxin11 to a termination codon (p.Q17X), leading to early termination of the synthesis of syntaxin11 and the loss of 240 amino acids in the proband. (B and C) The same heterozygous mutation was detected in her parents (B: sequence from her father; C: sequence from her mother).

A proband, female, aged 10 years and 3 months, with a medical history of recurrent fever and abdominal distention prior to the age of 5 years and 9 months, was admitted to our hospital with recurrent fever and cough for 3 days. Five years and nine months before admission to our hospital (when the child patient was 4 years and 6 months old), she was diagnosed with Epstein–Barr virus (EBV)–HLH due to fever, hepatosplenomegaly, pancytopenia, increase of ferritins and positive EBV-DNA, and thus was treated based on the HLH-2004 plan for 8 weeks. Treatment was then stopped, and the disease remained in continuous remission for 22 months. Three years and nine months before admission to our hospital, the patient was diagnosed as having a relapse of EBV–HLH due to the aforesaid symptoms and her positive blood EBV–DNA, and she was treated again by chemotherapy based on the HLH-2004 plan for 8 weeks. Symptoms relieved and she stopped taking drugs, and the duration of the disease remission reached about 19 months. Two years before admission to our hospital, the child patient developed recurrent fever and abdominal distension, which improved after receiving anti-infective therapy and taking dexamethasone (details not known) in a local clinic. She denied having a history of infectious disease and has been inoculated with hepatitis B vaccine, BCG vaccine, poliomyelitis attenuated live vaccine, diphtheria pertussis tetanus vaccine, measles attenuated live vaccine, and epidemic encephalitis B vaccine, without any abnormal reaction. The patient was born in full term, G3P3, her parents’ marriage was consanguineous (Fig. 1), and the two elder brothers respectively developed recurrent fever and abdominal distension symptoms similar to the patient's at the ages of 4 and 5 years and died of treatment failure in another hospital. Specific diagnosis and treatment as well as causes of death were unknown. After admission to our hospital, the child patient had a continuous high fever (39–40 °C), with an acute serious sickly appearance, without stained yellow skin or rash, but scattered ecchymosis was found. The superficial lymph nodes were not affected but her tonsils were slightly swollen. Medium and fine moist rales were heard in her right lung. Her abdomen was distended, the liver was 3.0 and 5.0 cm below the costal margin and the xiphoid, respectively. No abnormity was found in her nervous system. The hemogram revealed pancytopenia with the neutrophils 0.29 × 10^9^/L, Hb 52 g/L, and platelets 6 × 10^9^/L, respectively. The fibrinogen was decreased (the lowest value was 44 mg/dL), and the ferritin was elevated (995.3 ng/mL). Her triglyceride was normal, and a hemophagocytic phenomenon was found in a bone marrow examination. Multiple etiological examinations (blood culture, fungus G test and GM test, mycobacterium tuberculosis T-SPOT, mycoplasma, chlamydia and EBV-DNA [six repeated examinations], cytomegalovirus [CMV]-DNA, TORCH and seven combined examinations of respiratory viruses) were negative. According to eight HLH clinical diagnosis standards formulated by the International Histiocyte Society in 2004, the patient was clinically diagnosed as HLH. Due to a positive family history, in order to further specify the causes of the disease, we examined the activity of NK cells, the CD107a degranulation function of cytotoxic T lymphocytes and NK cells, as well as hereditary HLH-related gene mutations, and found that:

1. the activity of NK cells in the proband was reduced slightly, the CD107a degranulation function in cytotoxic T lymphocytes and NK cells also was obviously declined, and her parents also presented with reduced NK cell activity and slightly declined CD107a degranulation in CTL and NK cells (Fig. 2);
2. a homozygous mutation in *STX11* (c.49C>T), which converted the codon encoding the glutamine amino acid at position 17 in syntaxin11 to a termination codon (p.Q17X), leading to early termination of the synthesis of syntaxin11 and loss of 240 amino acids (Fig. 3).

This mutation was inherited from the parents of the proband (Figs. 1 and 3), and the result proved that the proband suffered from FHL-4. After admission to our hospital, the patient found relief after receiving treatment based on the HLH-2004 protocol; she has been alive for 6 years and 10 months since the onset, has stopped taking the dexamethasone and etoposide, but still takes cyclosporine orally to maintain treatment. She has undergone HLA matching and now is actively looking for a donor to undergo a hematopoietic stem cell transplantation.

## 3 Discussion

FHL is an autosomal recessively inherited disease arising from a defect of the cytotoxicity of CTL/NK cells (encapsidation, transfer, release, and other functions of cytotoxic granules), which mostly appears during childhood. Its incidence is about 1/50,000 liveborn infants, the majority of which lack a family history. The disease is generally manifested as fatal HLH and can be healed only after immunochemotherapy to control the disease and an hematopoietic stem cell transplantation.^[5]^ In FHL, FHL-2 accounts for about 20% to 50%, and FHL-3 accounts for about 25% of cases, but the ones arising from mutation of *STX11* only account for about 1%.^[2]^ Under normal conditions, the cytotoxicity of CTL/NK cells against target cells (such as B lymphocytes with an EBV mark on the surface after EBV infection, antigen-presenting cells, and tumor cells) is realized mainly by the Fas-L-induced cell apoptosis (non-secretory approach) and perforin-induced apoptosis and cytolysis of target cells (secretory approach); the latter is more important.^[6,7]^ When NK/CTL cells are stimulated by the antigens from abnormal cells and form an immunological synapse at the contact position with abnormal cells, the cytotoxic granules with perforin and granzyme B in the cells move toward the immunological synapse along a microtubule, and fuse with the cytomembrane at the position of the immunological synapse after sorting, maturation and priming, the granular contents are released by exocytosis (degranulation) into the cleft of the immunological synapse, and the perforin contained therein is inserted into the target cell membrane to form a pore canal. Subsequently, apoptosis and cytolysis of the target cells are induced through a change of the permeability of the target cells and granzyme B entering the target cells via the pore canal.^[2,8,9]^ The process involves a series of gene-encoding proteins and defects of those proteins would cause the perforin-induced apoptosis and cytolysis of the target cells to be deregulated, and failure to remove the target cells. Furthermore, the continuous stimulation of the antigens in the target cells causes the excessive proliferation and activation of CTL/NK cells (immunization is out of control), leading to a cytokine storm and further causing various clinical manifestations of hemophagocytic syndrome.^[2,8,9]^

The degranulation/exocytosis of CTL/NK cells is induced by soluble *N*-ethylmaleimide-sensitive factor attachment protein receptors (SNAREs), and the degranulation process is achieved by accurate coordinated regulation of various SNARE proteins, based on the specific binding of all members to SNAREs. SNAREs are divided into two groups: basic receptor proteins required for the exocytosis process and accessory proteins playing a regulatory role in the exocytosis process. The Syntaxin family belongs to the former while the Munc family, such as Munc18-1 and Munc18-2 (STXBP2), belongs to the latter. The basic receptor proteins can be further divided into two types according to their location: v-SNARE (vesicle-SNARE) located on the cytotoxic granular (vesicle) membrane, and t-SNARE (target-SNARE) located on the CTL/NK plasma membrane at the position of the immunological synapse. Syntaxin11 proteins belong to the latter. Positioned on chromosome 6q24, *STX11* contains two exons of which exon 2 encodes 287 amino acids, and is mainly expressed on the CTL and NK cell membranes of human beings.^[10]^ It is composed of an *N*-telopeptide, three-helix bundle (H_ABC_ structural domain) folded independently, a unispiral SNARE sequence motif, and a carboxyl terminal rich in cysteine. Unlike other proteins in the syntaxin family, because the carboxyl terminal of the syntaxin11 protein lacks a transmembrane domain, the Stx11 protein is anchored on the cytomembrane depending on isopentene group palmitoyl lipid, and it is therefore also known as lipid-anchored t-SNARE.^[11–14]^ When CTL/NK cells are stimulated and activated by a target cell with antigens, the cytotoxic granules (vesicles) therein move and get close to the plasma membrane at the position of the immunological synapse. The syntaxin11 (t-SNARE) on the plasma membrane combines with the v-SNARE-containing cytotoxic granules on the vesicle membrane (such as SNAP23, VAMP8, and VAMP2) to form a lipidic exchange channel, and specifically combines with Munc18-2/STXBP2, an accessory protein in SNAREs, to form a triplet complex, after which the vesicle membrane completely fuses with the plasmalemma to form a fusion pore and expand it, and the vesicular contents are released to complete the exocytosis.^[15,16]^

Mutation of *STX11* leads to the cytotoxic activity of CTL/NK cells becoming defective, and the FHL4 (OMIM605014) phenotype. In 2005, zur Stadt and others discovered a mutation of *STX11* for the first time in five FHL patients in a big Kurdish family from a consanguineous marriage.^[17]^ The investigators further discovered a homozygous mutation of *STX11* in the FHL patients in five other families from consanguineous marriages among Turkish and Kurdish people, and thus established FHL-4.^[17]^ Recent research has found that this mutation of *STX11* is not limited to Kurdish and Turkish people, but is found in other races.^[10,18–25]^ However, in Japan, 80% of the FHL cases result from a defect of *PRF1* and *UNC13D* s, without any reports on mutation of *STX11* yet,^[25]^ while mutation of *UNC13D* is the most common in Korea, with only one report on mutation of *STX11*^[24]^; there are reports on mutation of *STX11* in China.^[22,23]^

The proband mentioned in this report was born in a family from a consanguineous marriage (Fig. 1 Family Tree), and both of her elder brothers developed symptoms similar to hers and died at the age of 5 to 6 years. The child patient was infected with EBV at the age of 4 years and 6 months and HLH set in and she suffered from repeated attacks. In two previous attacks, she found relief for a long term after receiving the HLH-2004 protocol for 8 weeks (the duration of the remissions was 1 year and 10 months and 1 year and 7 months, respectively). As a result of *STX11* detection, a homozygous mutation of p.Q17X in the Stx11 protein appeared in the child patient, arising from each of her parents in the consanguineous marriage. This caused the loss of 270 of the 287 amino acids encoded for the Stx11 protein and the loss of the protein's function. We referred to the relevant literature and discovered that 11 different kinds of mutations of the Syntaxin11 protein have been reported at least currently: five missense/nonsense mutations, five minor deletions, and one large fragment deletion^[10,17–25]^; the mutation of Syntaxin11 in the proband mentioned in this study has not been reported in the literature yet, which is very rare for someone with FHL like our patient with such a serious defect of the Stx11 protein, late onset age (first onset at the age of 4 years and 6 months), relatively good response to preliminary therapy and long asymptomatic remission periods. According to Rudd^[18]^ and other reports, in four Turkish families resulting from consanguineous marriages with a mutation of *STX11*, the latest onset age was 7 years old (1–84 months). Having received short-term therapy and withdrawal of drugs, one patient from family A relapsed after a continuous and complete remission for 3 years; two patients from different families but with the same mutation (p.Q268X) had onset when they were 1 month and 7 years old, respectively. For four patients from two other families, families A and B with the same mutation (p.V124fs), the onset age was 3 to 39 months; for three sibling patients, the onset age was 3 months, 19 months, and 36 months, respectively. The cases of these patients, in combination with the family history, indicate that the phenotypic difference (individual difference) is large in patients with the same *STX11* genotype. Bryceson^[10]^ and others found that the NK cells in FHL-4 patients with newly isolated Stx11 defects developed a defect of the degranulation function when stimulated by activation signals. However, surprisingly, IL-2 could partially recover the degranulation function of CTL/NK cells in patients, according to the author, which may partially explain why patients with FHL-4 had a later onset age and longer remission periods without any specific treatment than that of patients with FHL-2 and FHL-3, but the mechanism is still unclear. According to the research of Hackmann and others,^[26]^ under normal conditions, the affinity between Munc18-2/STXBP2 and Syntaxin11 was 20 times higher than that between Munc18-2/STXBP2 and Syntaxin3. When Syntaxin11 was defective, CTL stimulated and activated by IL-2 enhanced the expression level of Syntaxin3, which could combine with Munc18-2/STXBP2 to partially recover the degranulation function of CTL cells; similarly, when Munc18-2/STXBP2 was defective, CTL stimulated and activated by IL-2 expressed Munc18-1, which could also combine with Syntaxin11 to partially recover the degranulation function of CTL cells. When the CTL/NK cells in patients with FHL-4 and FHL-5 are activated by IL-2, their degranulation function may be compensated and partially recovered by the respective combination between Syntaxin3 and Munc18-2/STXBP2 and between Syntaxin11 and Munc18-1. Based on reports that plasma EBV–DNA detections in another hospital were positive when the patient was attacked by HLH for the first and second times, while the results of 10 detections of EBV–DNA were all negative during the treatment in our hospital and at a follow-up of 1 year and 1 month, EBV in the plasma of the child patient might be cleared and HLH recurrence is unlikely to be induced by EBV. Our patient has an extremely serious Syntaxin11 defect (p.Q17X), but her onset age is late (4 years and 6 months) and the duration of the remission periods is long. The patient has been alive for 6 years and 10 months from the first onset of HLH till now (June 2018); therefore, we speculate that the compensation function mentioned above may be active in this patient.

At present, hematopoietic stem cell transplantation is the only method to radically cure FHL, but neither a proper donor in the China Marrow Donor Program nor a sibling donor (both of her elder brothers died) could be found for the patient, and her parents are p.Q17X heterozygote carriers with reduced CD107a degranulation and NK cell activity, so are not suitable as donors. Therefore, it is worthwhile to further deeply study the molecular mechanism and clinical application of IL-2 as a new adjuvant therapy for patients with FHL-4 and FHL-5 who have not yet found a suitable donor for hematopoietic stem cell transplantation.

## Acknowledgments

We thank the patient's family for their help and informed consent for publication of the case.

## Author contributions

**Conceptualization:** Qiang Li.

**Supervision:** Qiang Li.

**Validation:** Mingyan Jiang, Xue Tang.

**Writing – original draft:** Xia Guo.

**Writing – review & editing:** Qiang Li.

## References

[1] Henter JI Arico M Elinder G Familial hemophagocytic lymphohistiocytosis: primary hemophagocytic lymphohistiocytosis. Hematol Oncol Clin North Am 1998;12:417–33. 9561910 10.1016/s0889-8588(05)70520-7

[2] Rosado FG Kim S Hemophagocytic lymphohistiocytosis: an update on diagnosis and pathogenesis. Am J Clin Pathol 2013;139:713–27. 23690113 10.1309/AJCP4ZDKJ4ICOUAT

[3] Busiello R Adriani M Locatelli F Atypical features of familial hemophagocytic lymphohistiocytosis. Blood 2004;103:4610–2. 14739222 10.1182/blood-2003-10-3551

[4] Arico M Janca G Fischer A Hemophagocytic lymphohistiocytosis. Report of 122 children from the International Registry. FHL Study Group of the Histiocyte Society. Leukemia 1996;10:197–203. 8637226

[5] Henter JI Samuelsson-Horne A Arico M Treatment of hemophagocytic lymphohistiocytosis with HLH-94 immunochemotherapy and bone marrow transplantation. Blood 2002;100:2367–73. 12239144 10.1182/blood-2002-01-0172

[6] Kagi D Vignaux F Ledermann B Fas and perforin pathways as major mechanisms of T cell-mediated cytotoxicity. Science 1994;265:528–30. 7518614 10.1126/science.7518614

[7] Lowin B Hahne M Mattmann C Cytolytic T-cell cytotoxicity is mediated through perforin and Fas lytic pathways. Nature 1994;370:650–2. 7520535 10.1038/370650a0

[8] de Saint Basile G Ménasché G Fischer A Molecular mechanisms of biogenesis and exocytosis of cytotoxic granules. Nat Rev Immunol 2010;10:568–79. 20634814 10.1038/nri2803

[9] Luzio JP Hackmann Y Dieckmann NM The biogenesis of lysosomes and lysosome-related organelles. Cold Spring Harb Perspect Biol 2014;6:a016840. 25183830 10.1101/cshperspect.a016840 PMC4142962

[10] Bryceson YT Rudd E Zheng C Defective cytotoxic lymphocyte degranulation in syntaxin-11 deficient familial hemophagocytic lymphohistiocytosis 4 (FHL4) patients. Blood 2007;110:1906–15. 17525286 10.1182/blood-2007-02-074468 PMC1976360

[11] Hellewell AL Foresti O Gover N Analysis of familial hemophagocytic lymphohistiocytosis type 4 (FHL-4) mutant proteins reveals that S-acylation is required for the function of syntaxin 11 in natural killer cells. PLoS One 2014;9:e98900. 24910990 10.1371/journal.pone.0098900 PMC4049605

[12] Tang BL Low DY Hong W Syntaxin 11: a member of the syntaxin family without a carboxyl terminal transmembrane domain. Biochem Biophys Res Commun 1998;245:627–32. 9571206 10.1006/bbrc.1998.8490

[13] Prekeris R Klumperman J Scheller RH Syntaxin 11 is an atypical SNARE abundant in the immune system. Eur J Cell Biol 2000;79:771–80. 11139139 10.1078/0171-9335-00109

[14] Advani RJ Bae HR Bock JB Seven novel mammalian SNARE proteins localize to distinct membrane compartments. J Biol Chem 1998;273:10317–24. 9553086 10.1074/jbc.273.17.10317

[15] Spessott WA Sanmillan ML McCormick ME SM protein Munc18-2 facilitates transition of Syntaxin 11-mediated lipid mixing to complete fusion for T-lymphocyte cytotoxicity. Proc Natl Acad Sci USA 2017;114:E2176–85. 28265073 10.1073/pnas.1617981114 PMC5358394

[16] Müller ML Chiang SC Meeths M An N- terminal missense mutation in STX11 causative of FHL4 abrogates syntaxin-11 binding to Munc18-2. Front Immunol 2014;4:515. 24459464 10.3389/fimmu.2013.00515 PMC3890652

[17] zur Stadt U Schmidt S Kasper B Linkage of familial hemophagocytic lymphohistiocytosis (FHL) type-4 to chromosome 6q24 and identification of mutations in syntaxin 11. Hum Mol Genet 2005;14:827–34. 15703195 10.1093/hmg/ddi076

[18] Rudd E Göransdotter Ericson K Zheng C Spectrum and clinical implications of syntaxin 11 gene mutations in familial haemophagocytic lymphohistiocytosis: association with disease-free remissions and haematopoietic malignancies. J Med Genet 2006;43:e14. 16582076 10.1136/jmg.2005.035253 PMC2563216

[19] Marsh RA Satake N Biroschak J STX11 mutations and clinical phenotypes of familial hemophagocytic lymphohistiocytosis in North America. Pediatr Blood Cancer 2010;55:134–40. 20486178 10.1002/pbc.22499

[20] Horne A Ramme KG Rudd E Characterization of PRF1, STX11 and UNC13D genotype-phenotype correlations in familial hemophagocytic lymphohistiocytosis. Br J Haematol 2008;143:75–83. 18710388 10.1111/j.1365-2141.2008.07315.x

[21] Danielian S Basile N Rocco C Novel Syntaxin 11 gene (STX11) mutation in three argentinean patients with hemophagocytic lymphohistiocytosis. J Clin Immunol 2010;30:330–7. 19967551 10.1007/s10875-009-9350-4 PMC7370861

[22] Wang Y Wang Z Zhang J Genetic features of late onset primary hemophagocytic lymphohistiocytosis in adolescence or adulthood. PLoS One 2014;9:e107386. 25233452 10.1371/journal.pone.0107386 PMC4169386

[23] Wang JS Wang Z Wu L Etiology analysis of 38 patients with hemophagocytic syndrome. Zhongguo Shi Yan Xue Ye Xue Za Zhi 2010;18:1316–20. 21129284

[24] Sultanova AK Kim SK Lee JW A Novel Syntaxin 11 gene (STX11) mutation c.650T>C, p.Leu217Pro, in a Korean Child with familial hemophagocytic lymphohistiocytosis. Ann Lab Med 2016;36:170–3. 26709266 10.3343/alm.2016.36.2.170 PMC4713852

[25] Ishii E Hemophagocytic lymphohistiocytosis in children: pathogenesis and treatment. Front Pediatr 2016;13:47. 10.3389/fped.2016.00047 PMC4865497 27242976

[26] Hackmann Y Graham SC Ehl S Syntaxin binding mechanism and disease-causing mutations in Munc18-2. Proc Natl Acad Sci USA 2013;110:E4482–91. 24194549 10.1073/pnas.1313474110 PMC3839780

